# Conditional deletion of nonmuscle myosin II-A in mouse tongue epithelium results in squamous cell carcinoma

**DOI:** 10.1038/srep14068

**Published:** 2015-09-15

**Authors:** Mary Anne Conti, Anthony D. Saleh, Lauren R. Brinster, Hui Cheng, Zhong Chen, Shaleeka Cornelius, Chengyu Liu, Xuefei Ma, Carter Van Waes, Robert S. Adelstein

**Affiliations:** 1 Laboratory of Molecular Cardiology, NHLBI, Bethesda, MD 20814, USA; 2 Head and Neck Surgery Branch, NIDCD, Bethesda, MD 20892, USA; 3 Division of Veterinary Resources, Office of Research Services, NIH, Bethesda, Maryland 20892-5520, USA; 4 Transgenic Core Facility, NHLBI, NIH, Bethesda, MD, 20892, USA

## Abstract

To investigate the contribution of nonmuscle myosin II-A (NM II-A) to early cardiac development we crossed Myh9 floxed mice and Nkx2.5 cre-recombinase mice. Nkx2.5 is expressed in the early heart (E7.5) and later in the tongue epithelium. Mice homozygous for deletion of NM II-A (A^Nkx^/A^Nkx^) are born at the expected ratio with normal hearts, but consistently develop an invasive squamous cell carcinoma (SCC) of the tongue (32/32 A^Nkx^/A^Nkx^) as early as E17.5. To assess reproducibility a second, independent line of Myh9 floxed mice derived from a different embryonic stem cell clone was tested. This second line also develops SCC indistinguishable from the first (15/15). In A^Nkx^/A^Nkx^ mouse tongue epithelium, genetic deletion of NM II-A does not affect stabilization of TP53, unlike a previous report for SCC. We attribute the consistent, early formation of SCC with high penetrance to the role of NM II in maintaining mitotic stability during karyokinesis.

Nonmuscle myosin II-A (NM II-A) is a major cytoskeletal protein composed of a pair of heavy chains (230 kDa encoded by *Myh9*) and two pairs of light chains (20 kDa and 17 kDa). The protein plays an important role in early vertebrate development and genetic ablation of the heavy chain (NMHC II-A) results in lethality by embryonic day (E)6.5 due to a functional defect in the visceral endoderm^1^. Of particular note in the NM II-A ablated embryos were the defects in cell adhesion and the large irregularly-shaped nuclei present in the visceral endoderm. Enlarged nuclei and multinucleation have been noted before in the cardiac myocytes of NM II-B and NM II-C doubly ablated hearts^2^.

Since ablation of NM II-A or NM II-B leads to pre-natal lethality (E6.5 or E14.5 respectively) it became necessary to make use of conditional ablation using tissue specific expression of cre-recombinase to study the role of the various NM II paralogs in organ development. Therefore in order to examine the role of NM II-A in early cardiac development we made use of cre-recombinase under control of the Nkx2.5 promoter which is expressed in cardiac myocyte progenitors as early as E7.5. In addition it is also expressed in the tongue epithelium and in a mosaic pattern in the stomach and spleen^3^,^4^. Following conditional ablation of NM II-A, mice are born at the expected ratio but we failed to find any defect in cardiac development. However, as described in detail below, we consistently found development of squamous cell carcinoma (SCC) in the tongue of the ablated mice. The heterozygous mice were apparently normal.

It has recently been reported that NM II-A acts as a tumor suppressor of SCC^5^. An *in vivo* tissue-specific RNAi screen was used in which epithelial cell-specific delivery of lentiviral short hairpin RNA (shRNA) was performed in utero on pregnant females. Knocking out nonmuscle myosin heavy chain (NMHC) II-A induced metastatic SCC in the skin in epithelial specific keratin 14-cre conditional knockout mice (cKO) on a transforming growth factorβ-receptor-II (*TGFβ-R-II*) null background with a median latency of 3–7 mo. Moreover ablation of NM II-A in non-*TGFβ-R-II* compromised mice resulted in SCC of the skin after one year. As a mechanism for the onset of the tumor, it was also reported that in human and mouse keratinocyte cell lines NM II-A plays a role in regulating posttranscriptional stabilization of TP53. TP53 activation appeared to depend on NM II-A MgATPase activity since it was abolished by the NM II chemical inhibitor blebbistatin, and NM II-A appeared to be necessary for both TP53 stability and nuclear retention^5^.

Below we show that conditional loss of NM II-A alone in mouse tongue epithelium results in the development of SCC of the tongue. However the tumors we report on here appear during embryonic development, show a high degree of penetrance (100%), do not require combination with any other genetic alteration for induction and do not show alteration in TP53 stability and nuclear retention.

## Results

### Description of squamous cell carcinoma

**Figure 1:**
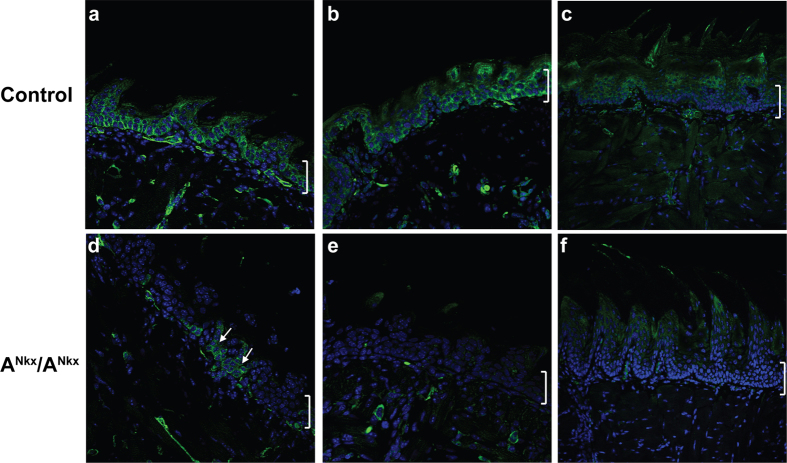
NM II-A expression levels decrease in A^Nkx^/A^Nkx^ mouse tongue epithelium. Although cre is expressed as early as E13, NM II-A (green) is still detected at low levels in some epithelial cells at P1 ((**d**), arrows, compared to control, (**a**)) as determined by immunofluorescence confocal microscopy. By P4, NM II-A is not seen in the tongue epithelium of the A^Nkx^/A^Nkx^ mouse ((**e**) compared to (**b**)). Expression of NM II-A decreases in the adult control tongue (**c**) and continues to be absent in the A^Nkx^/A^Nkx^ tongue. DAPI identifies nuclei (blue). Brackets on the right indicate tongue epithelium.

The original purpose in crossing NMHC II-A floxed mice and mice bearing cre-recombinase under control of the Nkx2.5 promoter was to investigate the contribution of NM II-A to early cardiac development. Thus the results reported here for the tongue were incidental to the negative cardiac findings (see below). Nkx2.5 is expressed early in cardiac development at E7.5. In addition it is also expressed in the tongue epithelium and in a mosaic pattern in the stomach and spleen^3^. For mice from the resulting crosses, the floxed Myh9 exon is designated A^flox^ and the floxed exon in the presence of cre is designated A^Nkx^. Tongue development from the floor of the pharynx is initiated at E11, and Nkx2.5-driven cre expression is detected as early as E13.5 in the tongue epithelium^3^. Nkx cre expression in the tongue epithelium was confirmed by crossing the Nkx cre mice to tdTomato mice^6^ which express red fluorescence in all tissues but in the presence of cre-recombinase, express GFP in place of tdTomato (Supplementary Fig. 1). Cre expression is detected at E14.5 in the tongue epithelium (Supplementary Fig. 1c, arrow), and a mosaic of NM II-A deletion is seen at postnatal day (P)1 in the A^Nkx^/A^Nkx^ mouse tongue epithelium (Fig. 1a,d, arrows, brackets indicate epithelium) but by P4, NM II-A is no longer seen (b,e). The deletion due to cre recombinase persists in the adult mouse as shown by the LacZ-linked assay shown in Supplementary Fig. 2. In accordance with this persistence, NM II-A is not detected in the A^Nkx^/A^Nkx^ tongue at later stages of development including the adult (Fig. 1f, compare to c).

A^Nkx^/A^Nkx^ mice, i.e. homozygous for the conditional NM II-A deletion, were born at the expected ratio (cross of A^flox^/A^flox^ to A^Nkx^/A^+^ gives approximately a 1:1:1:1 ratio or 26 A^Nkx^/A^Nkx^, 31 A^flox^/A^flox^, 23 A^Nkx^/A^+^ and 30 A^flox^/A^+^). The morphology of the heart and all other organs was normal. The A^Nkx^/A^Nkx^ heart shows no obvious abnormalities in stage of development and chamber morphology in embryos and adults compared to the control by histological analysis (Supplementary Fig. 3, n = 3). Surprisingly, the A^Nkx^/A^Nkx^ adult mice were 50% smaller than control littermates (A^Nkx^/A^+^, A^flox^/A^+^ or A^flox^/A^flox^) (n = 24 A^Nkx^/A^Nkx^ mice and n = 17 control mice, Supplementary Fig. 4).

**Figure 2:**
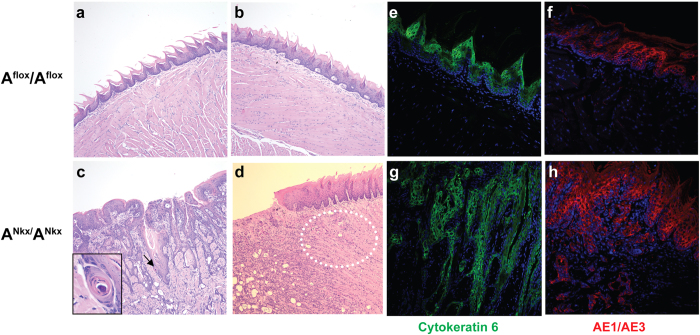
Tumors invade and differentiate in 6 week old mouse tongues. Normal control epithelium is shown (**a**,**b**,**e**,**f**) and examples of the tumors seen in A^Nkx^/A^Nkx^ mice (**c**,**d**,**g**,**h**), stained with H&E (**a**–**d**) and with antibodies to the SCC markers, cytokeratin 6 ((**e**,**g)** green) and AE1/AE3 ((**f**,**h**) red). The tumors form keratin pearls, evidence of differentiation within the tumor ((**c**) arrow and enlarged in inset) and invade the normal tongue musculature ((**d**) dotted oval). DAPI stains nuclei ((**e**–**h**) blue).) n = 47 A^Nkx^/A^Nkx^ mice.

**Figure 3:**
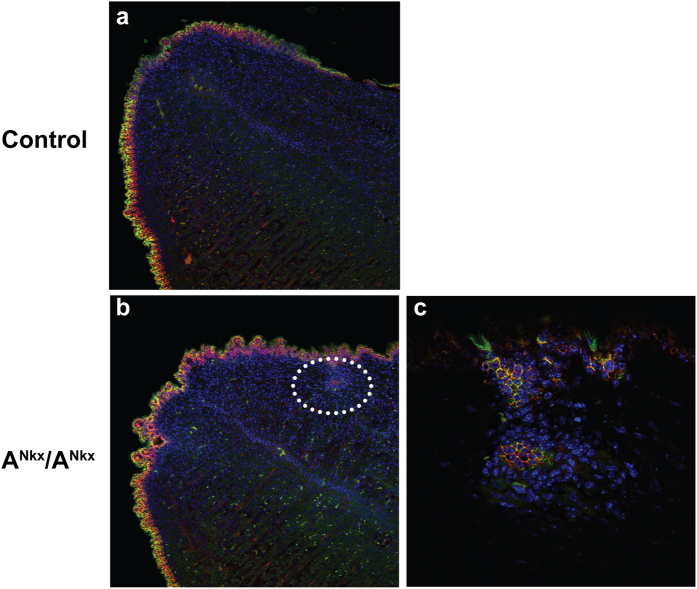
Tumor cells are detected at E17.5 in mouse tongue. Immunofluorescence microscopy of E17.5 control (**a**) and A^Nkx^/A^Nkx^ (**b**,**c**) mouse tongues. Cells have begun invading into the tongue musculature ((**b**) dotted oval, enlarged in (**c**)). Antibodies to NM II-C (green) and E-cadherin (red) are shown. DAPI stains nuclei (blue). n = 2 embryos.

**Figure 4:**
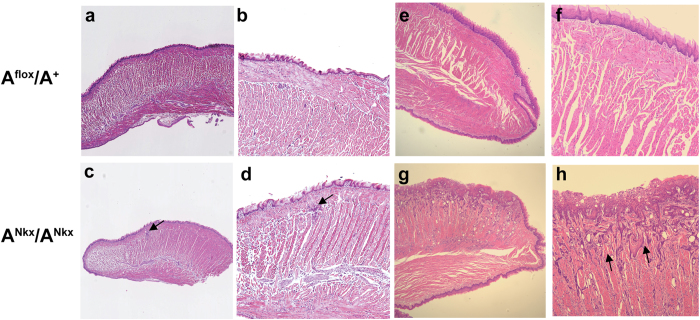
Tumors progress at P4 (a–d) and P30 (e–h). H&E stained sections of control (**a**,**b**,**e**,**f**) and A^Nkx^/A^Nkx^ (**c**,**d**,**g**,**h**) tongues indicate the extent and progression of tumor in the A^Nkx^/A^Nkx^ tongues. At P4 the tumor is still visible as foci in a section sagittal to the origin in the epithelium ((**c**,**d**) arrows). By P30 the tumor covers large areas of the tongue and is invading and streaming into the musculature ((**h**) arrows). P4 mice, n = 11; P30, n = 2.

Pathological examination revealed invasive SCC of the tongue which interfered with nutrition and in most cases starvation would have become fatal at approximately 8–12 weeks of age (mean = 10, range = 16, n = 21). For humane reasons the mice were sacrificed at the above ages. Figure 2 shows examples of SCC in the tongues of 6 week old mice. Of note are the keratin pearls that have formed within the tumor area (c, arrow and enlarged in inset) indicating evidence of squamous maturation or differentiation. Elsewhere the tumor is invading and replacing the normal musculature of the tongue (d, white dotted oval). Fig. 2e–h shows immunofluorescence stainings with the SCC markers cytokeratin 6 (e,g) and pan-keratin, AE1/AE3 (f,h) which are characteristic of SCC^7^. Within the length of time that the mice survived there was no evidence of metastases to the lymph nodes by microscopic examination (n = 3 A^Nkx^/A^Nkx^ mice). Figure 3 shows SCC in an E17.5 mouse tongue, the earliest time SCC was detected (b, white dotted oval, enlarged in c). By P1, NM II-A expression is decreased overall in the A^Nkx^/A^Nkx^ tongue epithelium and absent in groups of cells of the tongue epithelium (Fig. 1d). The progression of tumors from P4 to P30 is shown in Fig. 4. Tumors (c, arrow), began at one or several foci in the dorsal tongue epithelium, generally toward the front half of the tongue and invaded into the musculature (h, arrows). Some mice survived to 19 months of age but analysis of histological sections showed a tumor that was progressing more slowly with less extensive invasion of the underlying muscle (Supplementary Fig. 5c,d). Such slowly developing tumors may be due to variations in the extent or timing of cre expression in the tongue or due to the acquisition of additional genetic alterations.

### NM II paralog content in wild-type and cKO tongues

**Figure 5:**
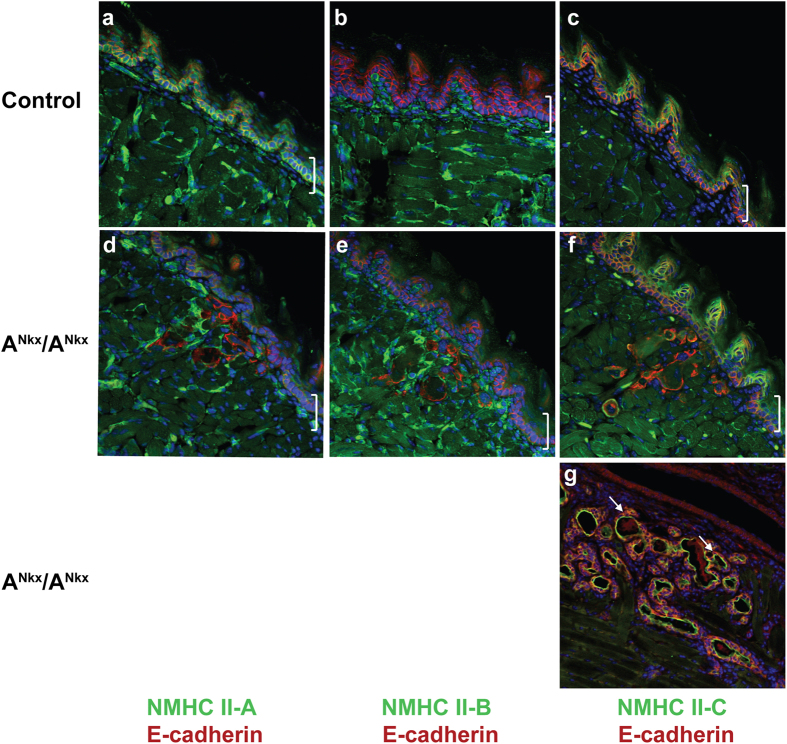
NM II paralogs II-A and II-C are detected in tongue epithelium. NM II-A (**a**) and NM II-C (**c**) are detected in the control tongue epithelium at P4 (green) while NM II-B is not (**b**). After deletion of NMHC II-A due to Nkx2.5-directed cre-recombinase activity, NM II-A is no longer detected in the tongue epithelium (**d**), II-B remains undetected (**e**) and II-C expression levels remain the same as in the control (**f**). Panel g shows the presence of NM II-C in the differentiated areas of the tumor (green, arrows). Tongues are co-stained with antibody to E-cadherin (red), DAPI indicates nuclei (blue). Brackets indicate tongue epithelium.

**Figure 6:**
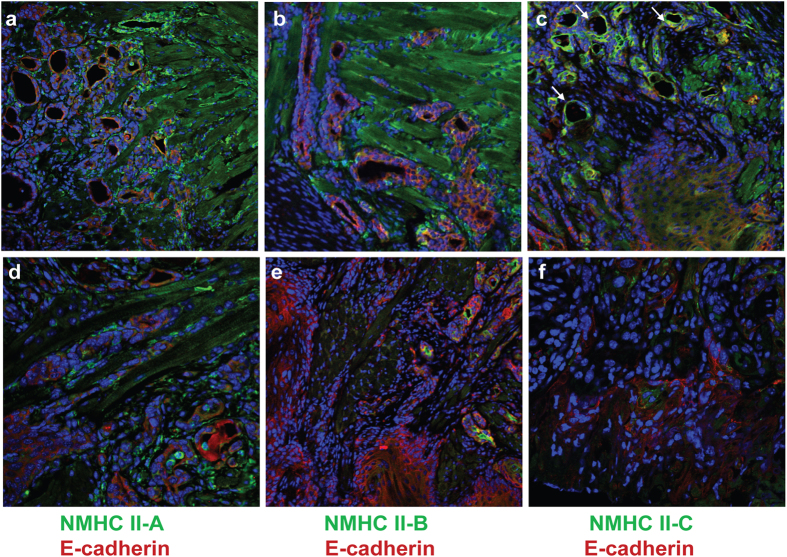
Migrating tumor cells. Expression of the NM II paralogs is undetectable in the migrating tumor cells that are invading the mouse tongue in the 6 week old A^Nkx^/A^Nkx^ mouse. This tumor is a grade 3, moderately to poorly differentiated tumor. Shown are two examples of immunofluorescence confocal microscopy of paraffin sections stained for NMHC II-A ((**a**,**d**) green), NMHC II-B ((**b**,**e**) green) or NMHC II-C ((**c**,**f**) green) and E-cadherin ((**a**–**f**) red). Sections shown in a-c are closer to the tongue surface while those in d-f are deeper into the muscle of the same tongue. Arrows in c indicate differentiated parts of the tumor which express II-C. DAPI indicates nuclei (blue).

To better characterize the wild-type and tumor cells, the content of NM II paralogs in the mouse tongue epithelium was assessed by mass spectroscopy of the isolated epithelial layer^2^,^8^. Although NMHC II-A (61% +/− 6 of total NM II content) and NMHC II-C (38% +/− 4) were detected, no NMHC II-B was detected by mass spectroscopy. This was confirmed by immunofluorescence microscopy which detected NM II-A (Fig. 5a) and NM II-C (c) but not NM II-B (b) in the epithelial layer of the control tongue where II-A and II-C colocalize with E-cadherin at cell-cell junctions (Fig. 5). In the A^Nkx^/A^Nkx^ tongue epithelium, NM II-A is no longer detected by immunofluorescence (d). There is no change in NM II-B or II-C expression in the epithelial layer in the A^Nkx^/A^Nkx^ tongue (Fig. 5e,f). Of note, in the invading tumor cells of the A^Nkx^/A^Nkx^ tongues, neither II-A, II-B nor II-C is detected, although II-C is present in differentiated portions of the tumor (Fig. 5g, arrows). The apparent loss of NM II-C may simply reflect its redistribution throughout the cell during migration and relocalization during differentiation. Figure 6 shows immunofluorescence micrographs of the tumor in the A^Nkx^/A^Nkx^ tongue showing that the invading tumor cells, unlike differentiated tumor cells, lack detectable levels of NM II-C, again most likely due to its diffuse distribution. The presence of II-C in the differentiated areas of the tumor is again indicated in Fig. 6c (arrows). There is no evidence for increased expression of either NM II-B or NM II-C in compensation for the loss of NM II-A^9^,^10^ in these tumor cells. H&E stain in a similar area of the tongue is shown in Supplementary Fig. 6. In addition, the actively migrating tumor cells do not express markers for mesenchymal cells typical of EMT including N-cadherin, smooth muscle actin and vimentin (Supplementary Fig. 7, circles).

### Reproducibility and crosses with other NM II mutant mice

**Figure 7:**
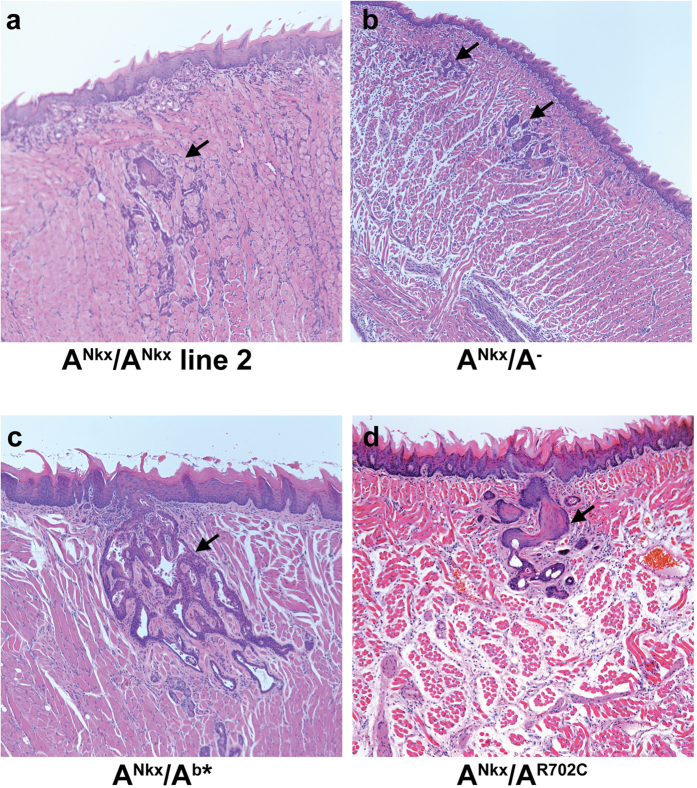
Tumors form in tongues from crosses to a second NM II-A floxed cell line, or to NM II-A^−^, NM II-A^b^*, or NM II-A^R702C^ mice. (**a**) Deletion of NM II-A using a second mouse line derived from NMHC II-A floxed embryonic stem cells results in a SCC (arrow) similar to that of the initial floxed mouse line. Crosses to mice which contain a null allele for II-A ((**b**), arrows), II-B in place of II-A ((**c**) arrow) or a point mutation, II-A R702C ((**d**), arrow) also produce SCC in the tongue epithelium although with varied timing of progression (see Table 1). H&E stained sections are shown.

In order to ascertain the reproducibility of the genesis of the SCC, a second, independent line of A^flox^ mice was derived from one of the original, heterozygous targeted embryonic stem cell clones. The homozygous floxed mice expressing cre in this second mouse line were also smaller than control littermates, developed SCC that was indistinguishable from that of mice derived from the original cell line and showed a similar timing of tumor formation and lethality (Fig. 7a, arrow).

Using targeted embryonic stem cells, we had previously reported the results of NM II-A ablation in mice. Although the heterozygous mice, which expressed one-half the wild-type amount of NM II-A were normal, the homozygous mice died at E6.5 due to a defect in the visceral endoderm^1^. To ascertain that there was no difference in the development of SCC due to the manner in which the deleted allele was introduced, A^Nkx^/A^+^ mice were crossed to NM II-A ablated heterozygous mice (A^+^/A^−^). Similar to A^Nkx^/A^+^ and A^+^/A^−^ mice, A^flox^/A^−^ mice were all normal with no evidence of SCC (n = 12 mice). However, similar to A^Nkx^/A^Nkx^ mice, A^Nkx^/A^**−**^ mice also developed a SCC in the tongue within 1-2 months (Fig. 7b, arrows; n = 5 mice). The phenotype of the A^Nkx^/A^**−**^ mice is indistinguishable from that of the A^Nkx^/A^Nkx^ mice in regard to NM II-A loss from the tongue epithelium, dorsal location of the tumor, timing of tumor progression and death of the mice, and pathological classification of the tumor as squamous cell carcinoma.

Thus it is the absence of expression of NM II-A in the tongue epithelium due either to cre-mediated deletion alone or in combination with a genetically null allele that leads to SCC.

Previous work has demonstrated that under certain conditions NM II-B can rescue defects in development due to the absence of NM II-A. cDNA encoding NMHC II-B was used to ablate NMHC II-A and thereby express NMHC II-B under control of the endogenous II-A promoter^11^. Of note we found that A^Nkx^/A^b*^mice in which NM II-A is deleted on one allele due to the activity of cre-recombinase and deleted on the second allele due to replacement with NMHC II-B develop an invasive, although less extensive SCC of the tongue but survive for up to 18 months (Fig. 7c, arrow; n = 9 of 9 mice) indicating that the expression of another paralog was ultimately unable to compensate for the loss of NM II-A. A^Nkx^/A^+^ mice were also crossed to NM II-A mice bearing a point mutation in NM II-A, R702C. Three of eight A^Nkx^/A^R702C^ mice developed a SCC in the tongue epithelium which was slow-growing and only identified on histological section at 8 months of age (Fig. 7d, arrow; see Table 1 for summary of these results including the tumor grade). Of note, A^+^/A^R702C^ mice and humans with mutations in NMHC II-A (including R702C) develop MYH9-related disease^12^,^13^,^14^,^15^ which is characterized by macrothrombocytopenia and glomerulosclerosis but not by tumor development.

### The role of TP53 in squamous cell tumorigenesis

**Figure 8:**
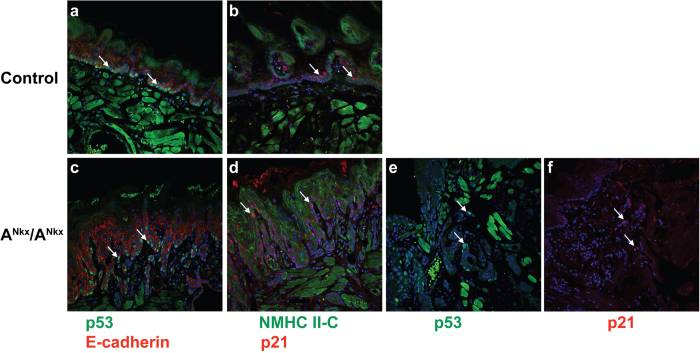
TP53 and p21 are expressed in γ-irradiated mouse tongues. A^Nkx^/A^Nkx^ and control mice were γ-irradiated (5 Gγ) and sacrificed 6 h later. Immunofluorescence microscopy indicates the presence of TP53 (green) in the nuclei in both control and A^Nkx^/A^Nkx^ tongues ((**a**,**c**,**e**) arrows). Antibodies to E-cadherin (red) stain cell-cell junctions in a, c. A target of TP53, p21 (red) is also present in nuclei of both control and A^Nkx^/A^Nkx^ tongues after γ-irradiation ((**b**,**d**,**f**) arrows). NMHC II-C ((**b**,**d**) green) is also shown. Sections in a-d are close to the original epithelial layer while sections in e,f are deeper within the tumor. Bright green signal in a,b,e is due to autofluorescence of muscle cells. DAPI stains nuclei (blue).

Previous work suggested that TP53 activation depended on NM II-A MgATPase activity since it was abolished by the NM II chemical inhibitor blebbistatin, and NM II-A appeared to be necessary for both TP53 stability and nuclear retention^5^. We investigated the possible role of TP53 in tumorigenesis in the mice generated here using two different methods. A^Nkx^/A^Nkx^ and control mice were γ-irradiated, sacrificed 3 or 6 hours later and sections of the tongue were stained for immunofluorescence microscopy. Figure 8 shows that at 6 hours post irradiation, both the A^Nkx^/A^Nkx^ and control mice sequestered TP53 in the nucleus after γ-irradiation (a,c,e, arrows). Additionally, both the A^Nkx^/A^Nkx^ and control mice showed an increased signal in the nucleus for p21, a target gene of TP53 expression (b,d,f, arrows). Similar results were seen at 3 hours post irradiation. Although TP53 and p21 expression were not generally observed in the non-γ-irradiated A^Nkx^/A^Nkx^ and non-γ-irradiated control tongues (Supplemental Fig. 8, dashed ovals), there were occasionally areas of faint p21 expression observed within the tumors. Supplementary Fig. 9a, (arrows) shows an example of this faint p21 signal in the absence of γ-irradiation (compare to p21 signal after γ-irradiation, Supplementary Fig. 9b).

The second method used to investigate the possible influence of TP53 on tumor development was an *in vitro* β–lactamase FRET reporter assay using a stably expressing TP53 reporter vector containing copies of the TP53 binding site cloned from the promoter of the p21 gene. The human SCC cell line UMSCC-74A was treated with control or NMHC II-A siRNA and subsequently incubated with etoposide which causes DNA damage and is expected to elicit a TP53 response in the nucleus. After 72 h, results indicate that siRNA transduction and knockdown of NMHC II-A itself elicited a TP53 reporter response and the presence of the TP53 inducing compound, etoposide increased that signal (Supplementary Fig. 10). These observations support an intact TP53 response.

### NMHC II and co-occurring genomic alterations in human head and neck SCC (HNSCC)

**Figure 9 f9:**
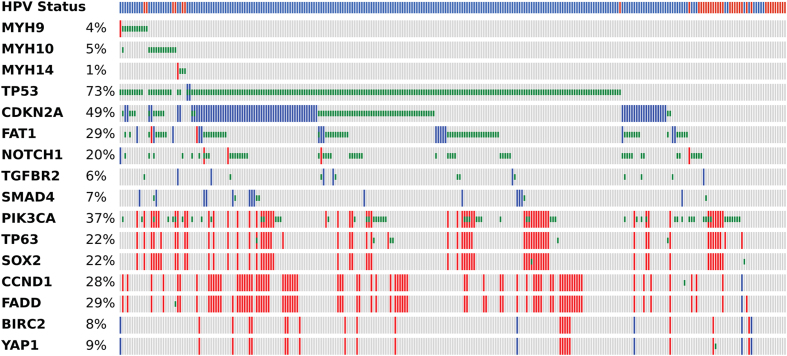
TCGA OncoPrint. Genetic alterations of MYH9, MYH10 and MYH14, co-occurrence with tumor suppressor and oncogenes in HNSCC tissues from TCGA dataset are shown. Genetic data of MYH family members, tumor suppressor genes, and oncogenes were extracted from TCGA HNSCC project through cBioPortal, and presented as OncoPrint for 279 HNSCC cases. Samples (n = 279) are displayed in columns and arranged to emphasize mutual exclusivity among mutations. Color coding indicates mutation type: red, homozygous amplification; blue, homozygous deletion; green, mutation. Left, mutation percentage. Top, HPV status.

The recently published Cancer Genome Atlas dataset^16^ for 279 HNSCC was analyzed for genomic alterations in NMHC II genes MYH9, 10 and 14, and for co-occurrence with human papilloma virus (HPV) status, selected mutations, and copy number alterations that are significant in HNSCC, as summarized by the Oncoprint in Fig. 9^17^,^18^. MYH9 and MYH10 mutations were identified at a similar frequency in 4–5% of cases, and MYH14 was mutated in 2 tumors, and these mutations tended to be mutually exclusive except in one tumor where co-occurrence of MYH9 and 10 mutations was observed. MYH9, 10 and 14 mutations occurred in both HPV-negative (HPV-) tumors, which were uniformly mutant for tumor suppressor TP53, and in HPV-positive (HPV+) tumors, with TP53 inactivation by the HPV E6 oncogene. MYH9, 10 and 14 mutant tumors also exhibited mutational inactivation and/or copy loss of commonly altered tumor suppressors CDKN2A (p16), FAT1, or NOTCH1, and infrequently altered TGFβ pathway components, TGF-B-R2 or SMAD4. Mutation or co-amplification of PI3K catalytic subunit PIK3CA with TP63 and SOX2 loci at 3q26, and amplification of CCND1 and FADD on 11p13, were found. Together, these observations reveal that NMHC II mutations in humans are associated with other genomic alterations characteristic of HPV− and HPV+ HNSCC.

## Discussion

Using Nkx2.5 promoter mediated expression of cre-recombinase we ablated NM II-A in the cardiac myocytes and the tongue. The A^Nkx^/A^Nkx^ mice were born at the expected ratios and did not appear to have any defects in heart development. This finding was of interest because NM II-A is expressed in addition to NM II-B in cardiomyocytes of the early heart tube. The lack of phenotype may reflect an instance of compensation of the II-B isoform for II-A in these cells. Later in development NM II-A is not seen in the cardiomyocytes.

Although we failed to observe any abnormalities in heart development, we were consistently able to produce SCC in the tongues of the affected mice (47/47 mice from two independently derived lines). Since cre expression in the tongue initiates at approximately E13.5, close to the initiation of tongue development (E11) and the earliest tongue tumors are detected at E17.5, we suspect that the early loss of NM II-A in the developing epithelial cells is important for tumor initiation. Both the high penetrance and early onset of the tumor are unusual features of this SCC mouse model^19^. Additionally, the cross of A^Nkx^/A^+^ mice to the A^+^/A^**−**^ mice indicated that the source of the deletion, either a conditional or a genetically null allele did not make a difference in the phenotype.

Our work appears to differ from the mice reported previously^5^ in the following: All of the homozygous A^Nkx^/A^Nkx^ mice developed SCC on a wild-type background with no other deletions or mutations and in a relatively short period of time. Moreover, as noted above, we failed to observe an effect of NM II-A loss on either TP53 stability or nuclear retention unlike that previously reported.

TCGA analysis reveals that NMHC II genes are mutated in ~10% of human HNSCC and are associated with a variety of common and less frequent genomic alterations implicated in development of these tumors. While introduction of MYH9 shRNA into murine embryos in a *TGFβ-R-II* deficient background promoted tumorigenesis and was linked to a defect in function of wild-type TP53^5^, mutation of MYH9 and other NMHC II genes in human HNSCC is invariably linked to TP53 mutation in HPV- or inactivation in HPV+ human tumors. Mutation of TGFβ-R-II or copy loss of Smad4 was observed in one tumor each and implicated as a genomic driver in 2/11 MYH9 mutant tumors, a frequency similar to that for these individual genes in the overall dataset. The co-occurrence of NM II mutations with TP53 mutations and other characteristic genomic copy number alterations affecting tumor suppressor or oncogene loci is consistent with genomic instability and multigenic pathogenesis in human tumors.

In the developing tongue epithelium where some cells still lack NM II-C (expression is only initiated at approximately E13.5) and all cells lack NM II-B, ablation of NM II-A could render these cells vulnerable to destabilization of the cytokinetic program. This destabilization could contribute to abnormal cell division and the genesis of the tumor we observe in these NM II-A cKO mice. We propose that the high penetrance of SCC that we have found in the NM II-A ablated mice is related to the roles that NM II has been shown to play in both cytokinesis and more specifically in karyokinesis^2^.

## Methods

### Mice

NMHC II-A^flox^ 20 (MMRRC #32096), NMHC II-A^b^* 11 (MMRRC #34321) and NMHC II-A^R702C^ 12 (MMRRC #36196) mice have been previously described. Nkx2.5 cre-recombinase mice were from Dr. Richard Harvey (Victor Chang Cardiac Research Institute) and tdTomato-GFP mice from Jackson Laboratories (#007576). All methods involving mice were carried out in accordance with the approved guidelines of the ACUC, NHLBI, NIH. All experimental protocols were approved by the ACUC, NHLBI, NIH.

For crosses of the A^Nkx^ line to A^**−**^ (NMHC II-A null allele), A^b^* (NMHC II-B in place of NMHC II-A), and A^R702C^ (NMHC II-A point mutant) lines, A^Nkx^/A^+^ mice were crossed to A^−^/A^+^, A^b^*/A^+^ or A^R702C^/A^+^ mice until the desired genotype was achieved. All of the above strains were generated by homologous recombination and are on a mixed background of 129Sv/C57BL6J. In the case of the A^R702C^ line, the neomycin cassette was removed by a cross to CMV-cre mice (Jackson Laboratories, #003465) which are on a BalbC background. The A^R702C^ line is therefore on a mixed 129Sv/C57BL6J/BalbC background.

### Immunofluorescence confocal microscopy

Mouse organs were fixed in 4% paraformaldehyde, embedded in paraffin and sectioned as previously described^21^. Frozen sections were prepared by fixing tissue in 4% paraformaldehyde for 40 min and transferring to 20% sucrose overnight at 4 °C. Immunofluorescence microscopy was performed using a Zeiss 510 LSM Meta or a Zeiss 780 confocal microscope.

### Antibodies

Antibodies to NMHC II-A (Covance, 1:1000) II-B (Covance, 1:3000) and II-C (Covance, 1:2000) were used for immunofluorescence staining. Antibodies to E-cadherin (BD Transduction Labs, 1:250), N-cadherin (Zymed, 1:250), p21 (BD Pharmingen, 1:50), TP53 (Leica NCL, 1:1000), smooth muscle actin (Sigma, 1:1000), and vimentin (Sigma, 1:40) were used at the dilutions indicated.

### β-galactosidase detection

A LacZ detection kit (InvivoGen, rep-lz-t) was used to detect cre-recombinase activity in frozen sections of mouse tissue.

### Gamma-irradiation

Mice were γ-irradiated (5 Gγs) in a Gammacell 40 Cesium irradiator and sacrificed after 3 or 6 hours. Tongues were excised and fixed in 4% paraformaldehyde as described above.[Fig f9]

### Assessment of TP53 activation following NMHC II-A siRNA knockdown

The human head and neck SCC cell line UM-SCC-74A was obtained from University of Michigan and cultured in MEM media (Gibco) with 10% FBS (Gibco), 1x NEAA (Gibco) and 100 U/mL Pen/Strep (Gibco).

A subline of UM-SCC-74A stably expressing a β-lactamase reporter gene under the control of the TP53 DNA response element was used. Stable cell line creation was performed as per manufacturer’s suggested protocol (Invitrogen Lit. No. O13404.pps). Briefly, transient transfection of the CellSensor® Lentiviral Vector, pLenti bsd—7xp53RE Bla (Invitrogen), was performed followed by antibiotic selection with blasticidin (Invitrogen) for two weeks. Cells that expressed high levels of β-lactamase following stimulation with 5 μM cisplatin (Sigma) for 24 hours were sorted by FACS and cultured following recovery. This sorted stock is referred to as UM-SCC-74A p53 RE and is maintained in media containing 5 μg/mL blasticidin.

To assess TP53 activation UM-SCC-74A p53 RE was seeded at 5 × 10^3^ cells per well in 100 μL of media in 96-well plates. Immediately after plating 10 pmols of siRNAs were transfected into UM-SCC-74A p53 RE using 0.3 μL of Lipofectamine*®* RNAiMAX (Invitrogen) in 10 μL of *Opti-MEM*® per well as per manufacturer’s instructions. Following 72 hour knockdown, transfection media was removed and TP53 activity was stimulated with 100 μL of fresh media without phenol red containing 25 ng/mL etoposide (Sigma) for 18 hours. To measure β-lactamase expression cells were stained with the FRET β-lactamase substrate CCF4-AM using the LiveBLAzer™-FRET B/G loading kit (Invitrogen) as per manufacturer’s protocol (Publication Number MAN0002771). Briefly 10 μL of 6.6 ng/μL CCF4-AM working solution was added to each well in 50 uL of media and incubated at room temperature for 2.5 hours. Following staining, blue coumarin (~450 nm) and FITC (~520 nm) fluorescence was read using a *VICTOR*™ *X2* 2030 Multilabel Plate Reader. Background fluorescence was corrected by reading wells containing CCF4-AM stain with no cells. Stimulated DAPI fluorescence signal was divided by FITC fluorescence signal to normalize for cell number/well.

## Additional Information

**How to cite this article**: Conti, M.A. *et al.* Conditional deletion of nonmuscle myosin II-A in mouse tongue epithelium results in squamous cell carcinoma. *Sci. Rep.* **5**, 14068; doi: 10.1038/srep14068 (2015).

## Supplementary Material

Supplementary Information

## Figures and Tables

**Table 1:** Summary of Mouse Lines and Tumor Phenotypes.

Genotype	Phenotype	AverageLife Span	TumorGrade	Gender
A^Nkx^/A^Nkx^ line 1	SCC 32/32*	2-3mos	3^a^	21M 11F
A^Nkx^/A^Nkx^ line 2	SCC 15/15	2-3mos	3^a^	7M 8F
A^Nkx^/A^−^	SCC 5/5	2-3mos	3^a^	2M 3F
A^Nkx^/A^+^	no tumor 12/12			
A^Nkx^/A^b*^	SCC 9/9	18 mos	2^b^	6M 3F
Control	no tumor 4/4			
A^Nkx^/A^R702C^	SCC 3/8	8mos	2^c^	1M2F/1M7F
Control	no tumor 6/6			

^*^Number of affected mice per number of mice examined by H&E stained sections.

^a^Moderately to poorly differentiated.

^b^Moderately to well-differentiated.

^c^Moderately differentiated.
