# Supplementary material for: Conditional deletion of nonmuscle myosin II-A in mouse tongue epithelium results in squamous cell carcinoma
Source: Sci Rep. 2015 Sep 15;5:14068. doi: 10.1038/srep14068 (PMC4572924; doi:10.1038/srep14068)
Supplement: Supplementary Information [file srep14068-s1.doc]

**Supplementary Information**

Conditional deletion of nonmuscle myosin II-A in mouse tongue epithelium results in squamous cell carcinoma

Mary Anne Conti, Anthony D. Saleh, Lauren R. Brinster, Hui Cheng, Zhong Chen, Shaleeka Cornelius, Chengyu Liu, Xuefei Ma, Carter Van Waes, Robert S. Adelstein


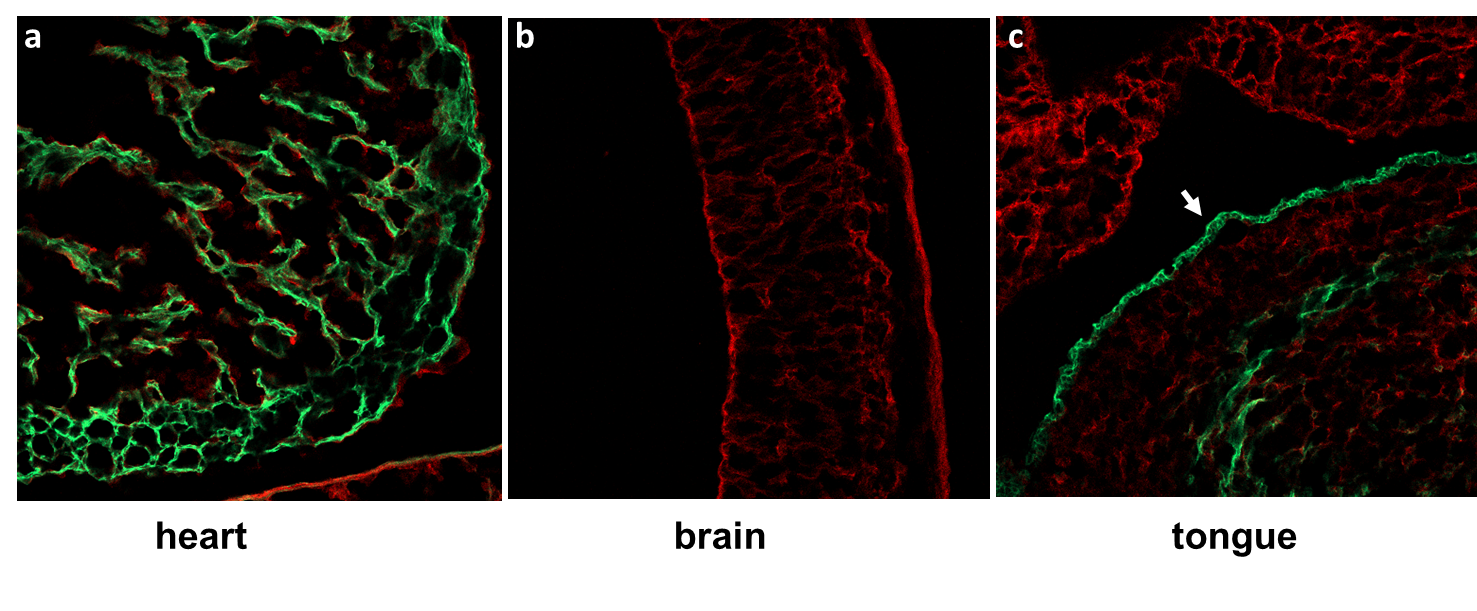


**Supplementary Figure 1. Confirmation of cre-recombinase expression in tongue epithelium.** A cell membrane-targeted tdTomato-GFP mouse embryo shows expression of cre-recombinase in (a) cardiomyocytes and (c) tongue epithelium (arrow) but not in (b) brain in cryosections of an E14.5 mouse embryo. Green color due to GFP expression replaces tdTomato signal (red) confirming cre-recombinase activity. Images from confocal microscopy are shown.

**
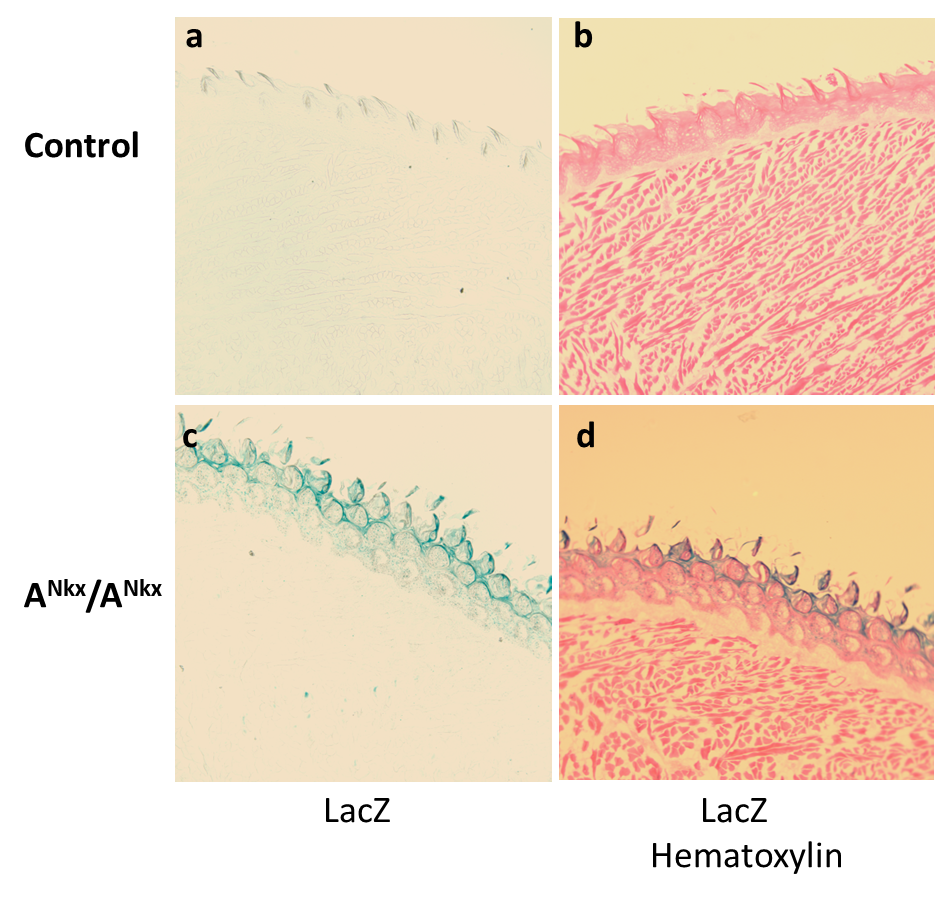
**

**Supplementary Figure 2. Persistance of cre-recombinase mediated deletion in the adult tongue epithelium.** Nkx2.5 cre recombinase mice also contained a β-galactosidase expression cassette with a 5’ floxed stop sign. Removal of the stop in the presence of cre permits expression of β-galactosidase which is detected in the presence of X-Gal as a blue stain in positive cells. A control mouse tongue stained for β-galactosidase is shown in a and with hematoxylin counter stain in b. The A^Nkx^/A^Nkx^ mouse tongue stained for β-galactosidase is shown in c and with hematoxylin counter stain in d.

**
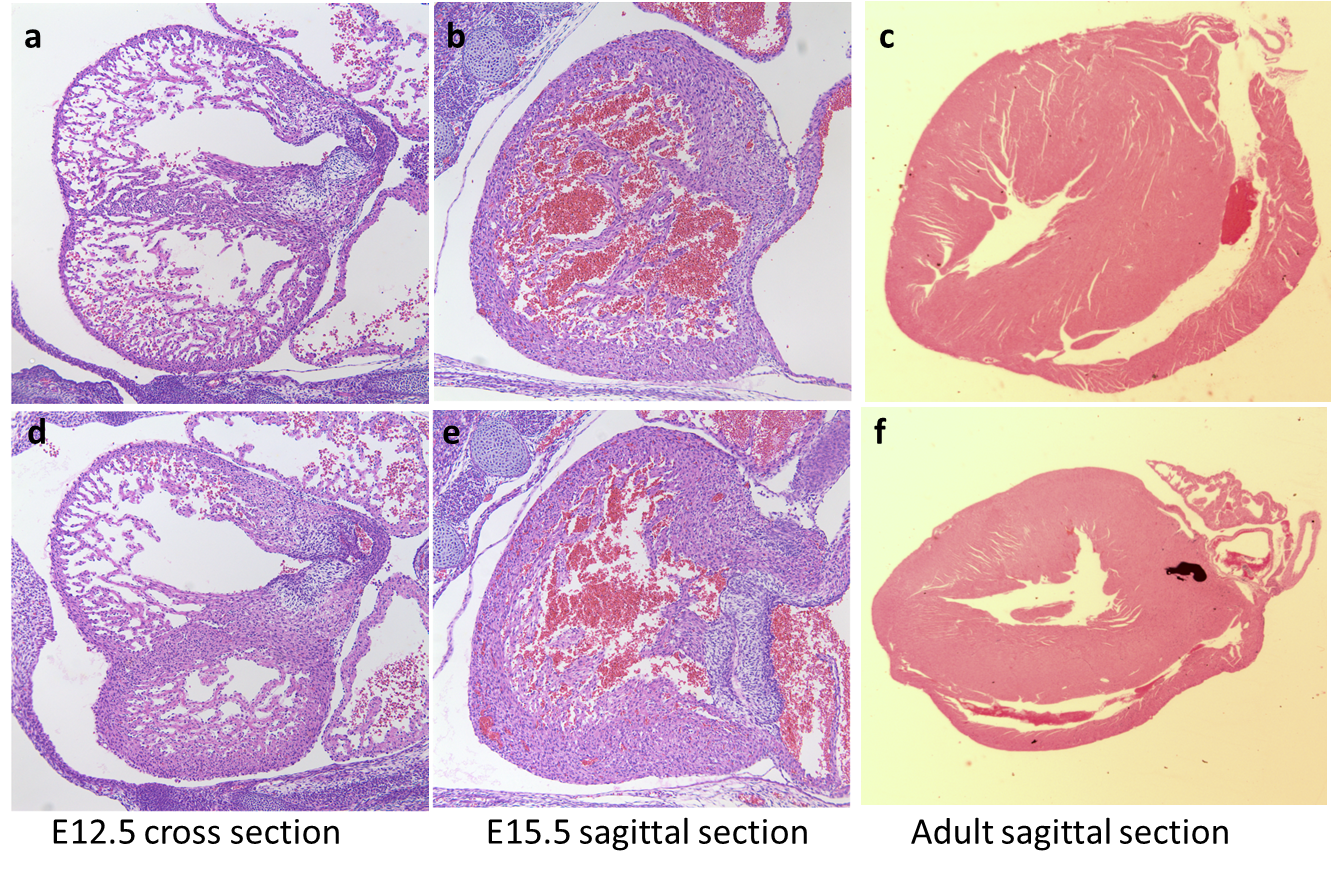
**

**Supplementary Figure 3. Normal morphology in A^Nkx^ /A^Nkx^ hearts.** H&E stained sections of E12.5 (a,d), E15.5 (b,e) and adult (c,f) hearts are shown for control (a-c) and A^Nkx^ /A^Nkx^ mice (d-f). The stage of development and morphology appear unaffected by the early deletion of NM II-A (E7.5) in the cardiomyocytes.

**
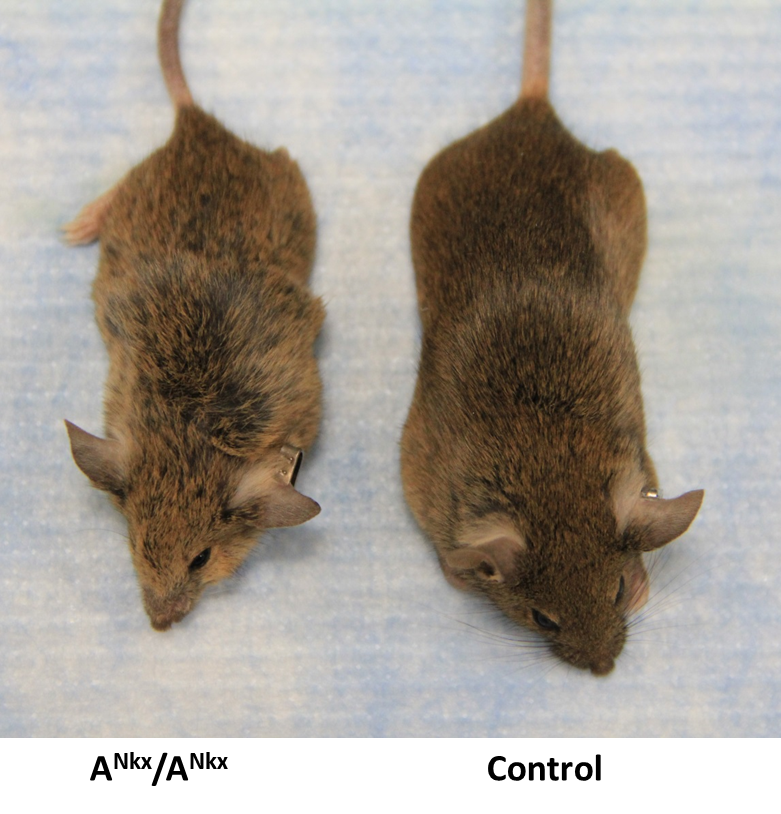
**

**Supplementary Figure 4. A^Nkx^ /A^Nkx^ mice are smaller than control littermates.** Smaller size of A^Nkx^/A^Nkx^ mice is due to lack of nutrition. Three month old A^Nkx^/A^Nkx^ (left) and control (A^Nkx^/A^+^, right) mice. Mice weighed 10.1 and 19.9g respectively.

**
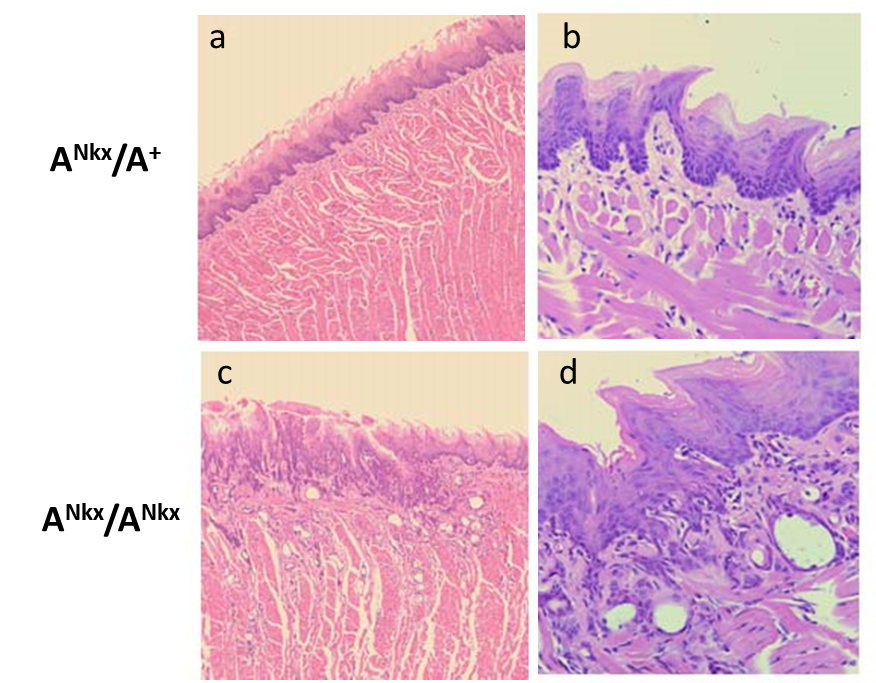
**

**Supplementary Figure 5. A^Nkx^/A^Nkx^ escaper mice.** H&E stained sections of control (a,b) and A^Nkx^/A^Nkx^ (c,d) mouse tongues at 18 months. A few A^Nkx^/A^Nkx^ mice survive longer than the average 2-3 months and show a more focal tumor which has barely begun to invade the musculature of the tongue (c).

**
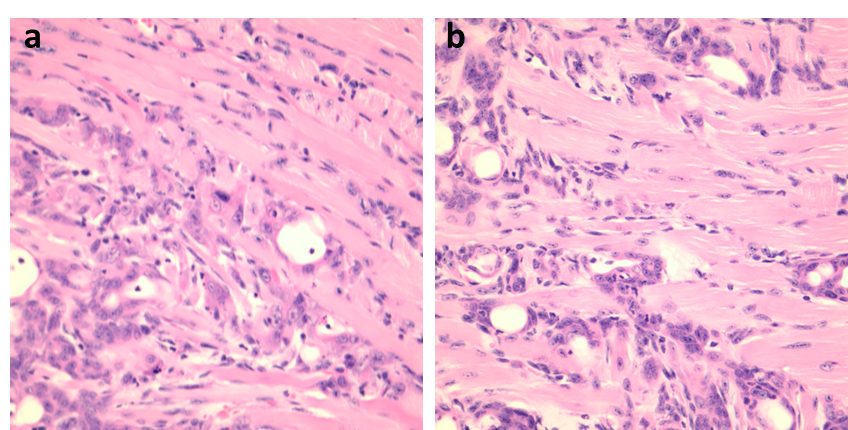
**

**Supplementary Figure 6. H&E stained sections from A^Nkx^/A^Nkx^ mouse SCC.** Examples of the area of immunofluorescence stained sections shown in Fig. 6 are presented in a and b.

**
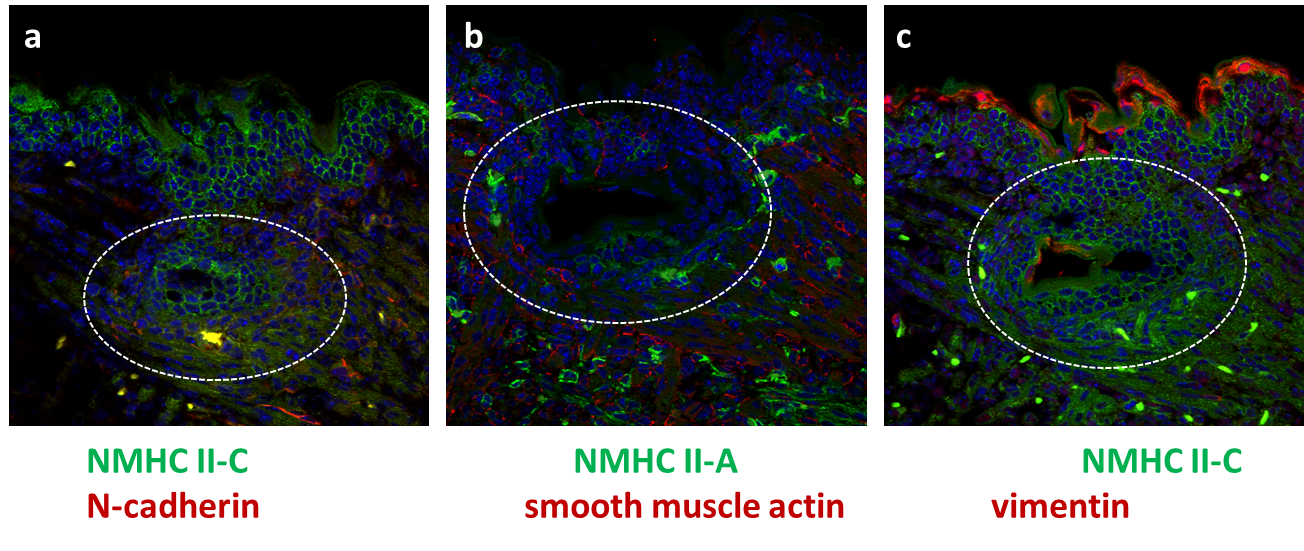
**

**Supplementary Figure 7. A^Nkx^/A^Nkx^ tumor stained for EMT markers.** Immunofluorescence confocal microscopy of sections of E17.5 A^Nkx^/A^Nkx^ mouse tongues stained for N-cadherin (a, red), smooth muscle actin (b, red) or vimentin (c, red) and co-stained for NMHC II-C (a,c, green) or NMHC II-A (b, green). There is some vimentin staining in the outermost layer of the epithelium and again within the differentiated area of the tumor but not in the migrating tumor cells surrounding the area of differentiation. Dashed ovals surround the principal area of the tumor. DAPI indicates nuclei (blue).

**
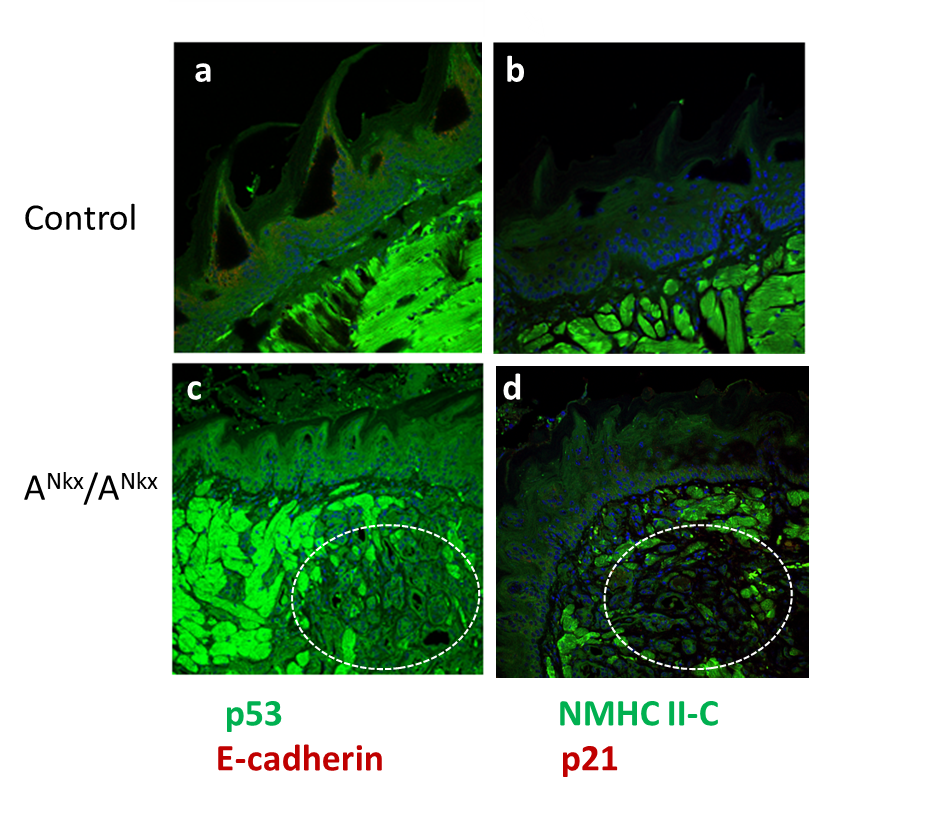
**

**Supplementary Figure 8. Control and A^Nkx^/A^Nkx^ mouse tongues from non-γ- irradiated mice.** Sections from control (a,b) and A^Nkx^/A^Nkx^  (c,d) mouse tongues stained for p53 (a,c, green) and p21 (b,d, red) are shown. These mice were not subject to γ-irradiation. Note the absence of p53 and p21 stain in the control and tumor samples. Areas of tumor are indicated by dashed ovals (c.d) and are dark compared to the bright autofluorescence from muscle cells.

**
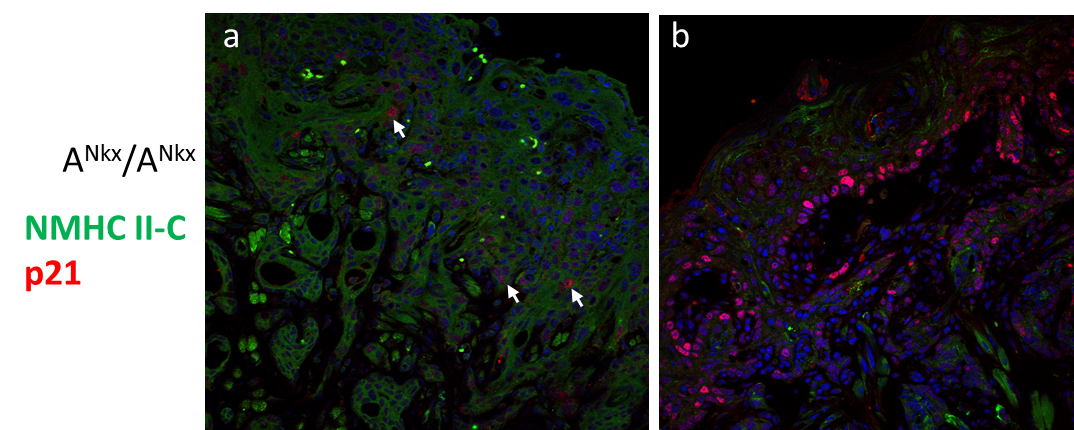
**

**Supplementary Figure 9. p21 expression in A^Nkx^/A^Nkx^  mouse SCC in the absence of γ-irradiation.** Sections of tumors in A^Nkx^/A^Nkx^  mouse tongues were examined by immunofluorescence confocal microscopy. p21, a downstream target of TP53, is detectable in some sections even without γ-irradiation (a, red, arrows) although at very low levels. The level of p21 signal can be compared to that seen after γ-irradiation (b, red). NMHC II-C, green; DAPI indicates nuclei (blue).

**
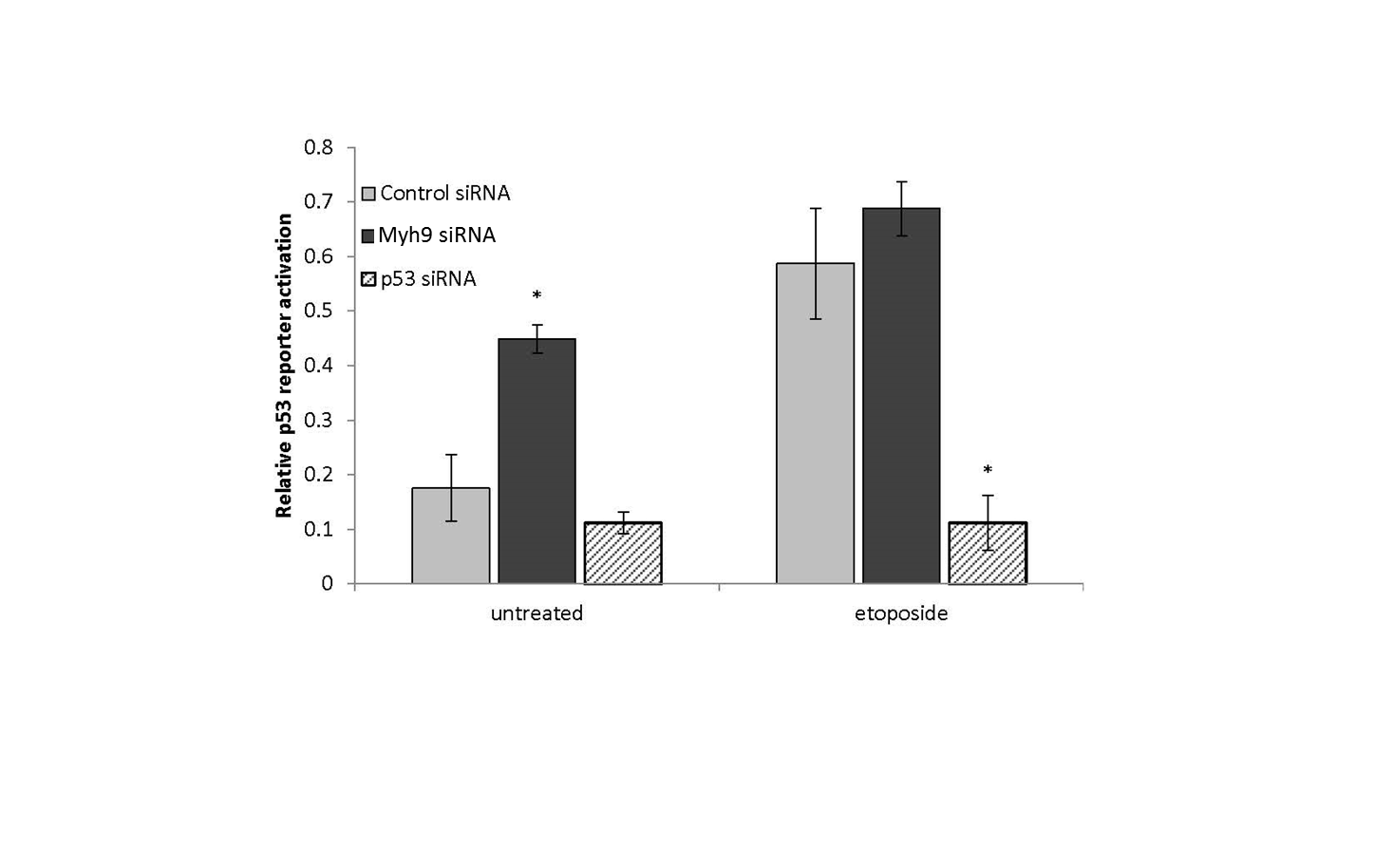
Supplementary Figure 10. FRET assay for TP53 activity.** Stable cell line UM-SCC-74A p53 RE was transfected for 72 hrs with the indicated siRNAs. TP53 activation was stimulated by 18-hr treatment with etoposide before assay. Data represents the mean of at least 3 experimental replicates and error bars represent SEM. * denotes a p-value < 0.05 by two-tailed Student’s t-test when compared to control siRNA for each condition
